# The Role of Systemic Immune-Inflammation Index (SII) in Diagnosing Pediatric Acute Appendicitis

**DOI:** 10.3390/diagnostics15151942

**Published:** 2025-08-02

**Authors:** Binali Firinci, Cetin Aydin, Dilek Yunluel, Ahmad Ibrahim, Murat Yigiter, Ali Ahiskalioglu

**Affiliations:** 1 Department of Pediatric Surgery, Medical Faculty of Ataturk University, 25100 Erzurum, Turkey; drcetinaydin@yahoo.com.tr (C.A.); yunlueldilek@gmail.com (D.Y.); ehmedri051@gmail.com (A.I.); dryigiter@gmail.com (M.Y.); 2 Department of Anesthesiology and Reanimation, Medical Faculty of Ataturk University, 25100 Erzurum, Turkey; aliahiskalioglu@hotmail.com

**Keywords:** acute appendicitis, systemic immune-inflammatory index, pediatrics

## Abstract

**Background and Objectives**: Accurately diagnosing acute appendicitis (AA) in children remains clinically challenging due to overlapping symptoms with other pediatric conditions and limitations in conventional diagnostic tools. The systemic immune-inflammation index (SII) has emerged as a promising biomarker in adult populations; however, its utility in pediatrics is still unclear. This study aimed to evaluate the diagnostic accuracy of SII in distinguishing pediatric acute appendicitis from elective non-inflammatory surgical procedures and to assess its predictive value in identifying complicated cases. **Materials and Methods**: This retrospective, single-center study included 397 pediatric patients (5–15 years), comprising 297 histopathologically confirmed appendicitis cases and 100 controls. Demographic and laboratory data were recorded at admission. Inflammatory indices including SII, neutrophil-to-lymphocyte ratio (NLR), and platelet-to-lymphocyte ratio (PLR) were calculated. ROC curve analysis was performed to evaluate diagnostic performance. **Results**: SII values were significantly higher in the appendicitis group (median: 2218.4 vs. 356.3; *p* < 0.001). SII demonstrated excellent diagnostic accuracy for AA (AUROC = 0.95, 95% CI: 0.92–0.97), with 91% sensitivity and 88% specificity at a cut-off > 624. In predicting complicated appendicitis, SII showed moderate discriminative ability (AUROC = 0.66, 95% CI: 0.60–0.73), with 83% sensitivity but limited specificity (43%). **Conclusions**: SII is a reliable and easily obtainable biomarker for diagnosing pediatric acute appendicitis and may aid in early detection of complicated cases. Its integration into clinical workflows may enhance diagnostic precision, particularly in resource-limited settings. Age-specific validation studies are warranted to confirm its broader applicability.

## 1. Introduction

Acute appendicitis (AA) is one of the most common surgical emergencies in the pediatric population, often presenting as a primary cause of acute abdominal pain in pediatric surgery departments [1]. Despite significant advancements in medical diagnostics, accurately diagnosing AA in children remains a challenge. The clinical presentation may be unclear, with symptoms coinciding with other common childhood illnesses, making it difficult to distinguish appendicitis from less serious conditions [2]. Delayed or missed diagnoses of appendicitis can lead to severe complications, such as perforation, abscess formation, and peritonitis, which not only increase morbidity but also prolong hospital stays and increase healthcare costs [3]. Therefore, there is a pressing need for reliable, cost-effective, and easily accessible biomarkers that can aid in the early and accurate diagnosis of AA.

In recent years, there has been increasing interest in systemic inflammatory markers derived from routine blood tests, such as the neutrophil-to-lymphocyte ratio (NLR), platelet-to-lymphocyte ratio (PLR), and systemic immune-inflammation index (SII). These markers have shown promise in various inflammatory and oncological conditions, offering insights into disease severity and prognosis [4,5]. Among these, the SII, which combines platelet, neutrophil, and lymphocyte counts into a single index, has gained attention for its ability to provide a more comprehensive reflection of systemic inflammation compared to traditional markers like C-reactive protein (CRP) or white blood cell (WBC) count [6]. The SII is calculated using a simple formula, (neutrophil count × platelet count)/lymphocyte count, making it an attractive option for clinical use due to its ease of calculation and cost-effectiveness.

According to the current literature, acute appendicitis is commonly classified into uncomplicated and complicated subtypes based on clinical evaluation and intraoperative findings. The World Society of Emergency Surgery characterizes complicated appendicitis by the presence of gangrene, necrosis, or perforation, while uncomplicated forms are restricted to inflammation and hyperemia without transmural involvement or rupture. Despite the advancements in diagnostic modalities and novel biomarkers, no single biomarker currently provides consistently high sensitivity and specificity for differentiating these forms preoperatively. Therefore, attention has increasingly shifted to hematologic indices such as the NLR, PLR, and SII, which offer cost-effective and accessible indicators of systemic inflammation and immune response in pediatric appendicitis.

The SII has been extensively studied in adult populations, particularly in the context of cancer and cardiovascular diseases, where it has demonstrated strong predictive value for disease outcomes [7,8]. However, its application in pediatric AA remains limited. This gap in research is significant because children often present with atypical symptoms and are more susceptible to rapid disease progression, making timely and accurate diagnosis critical [9]. A reliable biomarker like the SII could serve as a valuable adjunct to clinical evaluation and imaging studies, potentially reducing the rate of negative appendectomies and improving patient outcomes.

Recent studies have shown that elevated SII levels are associated with more severe forms of AA and complications such as perforation and abscess formation in adult populations [10,11]. These findings suggest that the SII could be a useful tool not only for diagnosing AA but also for identifying patients at higher risk of complications. However, data on its efficacy in pediatric AA are limited, and further research is needed to validate its utility in this specific population. This study aims to investigate the diagnostic accuracy of the SII in pediatric patients with AA.

## 2. Materials and Methods

The study was started following approval from the Atatürk University local ethics committee (Approval No: B.30.2ATA.0.01.00/186, Date: 28 February 2025). It included patients aged 5 to 15 years who were diagnosed with acute appendicitis and underwent appendectomy at Erzurum Atatürk University Department of Pediatric Surgery Clinic between January 2020 and January 2025. Only patients for whom complete blood count (CBC) and other laboratory parameters were recorded in the hospital automation system during emergency admission and surgical preparation were included. Patient files were retrospectively reviewed, and data from CBC and laboratory analyses were collected.

The control group consisted of age- and sex-matched patients who underwent elective non-inflammatory surgical procedures such as circumcision, inguinal hernia repair, or umbilical hernia. Patients diagnosed with acute appendicitis based on physical examination, ultrasonography, or abdominal CT and who subsequently underwent surgery were included in the study. Exclusion criteria for both the patient and control groups include recent antibiotic therapy within the past two weeks, the presence of acute or chronic infectious diseases (febrile or afebrile), autoimmune disorders, chronic liver disease, malignancy, a history of medical or surgical interventions within the past three months, endocrine disorders, hematological or coagulation disorders that could affect CBC parameters, steroid use, a history of radiotherapy or chemotherapy, chronic gastrointestinal inflammatory conditions such as Crohn’s disease or ulcerative colitis, and the absence of recorded CBC results in hospital files.

Data collected included CBC parameters and C-reactive protein (CRP) levels. The study groups were compared in terms of age, sex, white blood cell (WBC) count, neutrophil count, lymphocyte count, platelet count, NLR, PLR, systemic immune-inflammation index (SII), and CRP levels. The SII index was calculated using the following formula: SII = [(neutrophil count × platelet count)/lymphocyte count]. In both groups, hematological and biochemical parameters obtained at the time of hospital admission were analyzed. While histopathological confirmation of appendicitis was obtained for all surgical cases, the classification into complicated and uncomplicated appendicitis was based on intraoperative and clinical findings.

### Statistical Analysis

All statistical analyses were performed using IBM SPSS Statistics for Windows, version 27.0 (IBM Corp., Armonk, NY, USA). Descriptive statistics were calculated for all study variables. Continuous data were expressed as either mean ± standard deviation (SD) for normally distributed variables or median with interquartile range (IQR) for non-normally distributed variables, as determined with Shapiro–Wilk tests and visual inspection of histograms. Categorical variables were summarized as counts or percentages. Between-group comparisons were conducted using Student’s *t*-test for normally distributed data and the Mann–Whitney U test for variables not conforming to normality. Chi-square or Fisher’s exact tests were applied to compare categorical variables, depending on expected cell counts.

To evaluate the diagnostic performance of the SII, PLR, and NLR in predicting acute and complicated appendicitis, receiver operating characteristic (ROC) curve analysis was employed. The area under the ROC curve (AUROC) was calculated to quantify discriminative ability. In addition, sensitivity, specificity, positive predictive value (PPV), and negative predictive value (NPV) were determined at optimal cut-off points, which were identified using Youden’s Index. A two-sided *p*-value < 0.05 was considered statistically significant.

## 3. Results

A total of 550 pediatric patient records were initially screened for eligibility. After applying the predefined inclusion and exclusion criteria, 153 patients were excluded due to incomplete laboratory data, recent infections, antibiotic use, chronic inflammatory or hematologic conditions, or missing records. Consequently, 397 pediatric patients were included in the study, comprising 297 patients diagnosed with acute appendicitis and 100 healthy controls. Among the appendicitis group, 78 patients (26.3%) had histologically confirmed complicated appendicitis.

As shown in Table 1, most inflammatory markers, including WBC, neutrophil count (NEU), CRP, NLR, PLR, and SII, were significantly elevated in the appendicitis group compared to controls (*p* < 0.001 for all). Apart from the diagnostic indices, several laboratory parameters also exhibited statistically significant differences between groups. Monocyte counts were markedly higher in the appendicitis group (median: 0.92 × 10^3^/µL vs. 0.54 × 10^3^/µL, *p* < 0.001), while eosinophil and lymphocyte counts were significantly lower (eosinophils: 0.02 vs. 0.13 × 10^3^/µL; lymphocytes: 1.67 vs. 3.13 × 10^3^/µL; both *p* < 0.001), reflecting an intensified systemic inflammatory response. Furthermore, CRP levels showed a substantial increase in appendicitis patients (median: 32.90 mg/L vs. 1.12 mg/L, *p* < 0.001), consistent with acute inflammation. Platelet counts, while statistically different (*p* = 0.043), showed overlapping ranges. No significant differences were observed in hemoglobin, hematocrit, or basophil counts. Notably, age and sex distributions were similar between groups, eliminating potential demographic confounders. In terms of clinical outcomes, duration of hospitalization was significantly prolonged in the appendicitis group (median: 3 vs. 2 days, *p* < 0.001).

**Figure 1:**
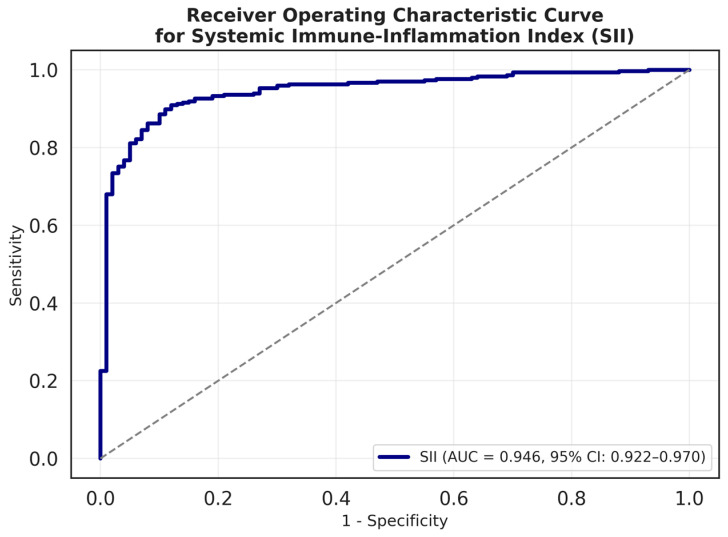
ROC curve for systemic immune-inflammation index in detecting appendicitis among pediatric patients.

The diagnostic performance of SII, NLR, and PLR in differentiating appendicitis from controls is summarized in Table 2. Among all indices, SII demonstrated the highest area under the ROC curve (AUROC = 0.95, 95% CI: 0.92–0.97), followed closely by NLR (AUROC = 0.96, 95% CI: 0.94–0.98). At an optimal cut-off value of >624, SII achieved 91% sensitivity, 88% specificity, PPV of 0.96, and NPV of 0.76. The corresponding ROC curve is presented in Figure 1. PLR, although statistically significant, yielded a lower AUROC of 0.77 (95% CI: 0.72–0.81), limiting its standalone diagnostic utility.

**Figure 2:**
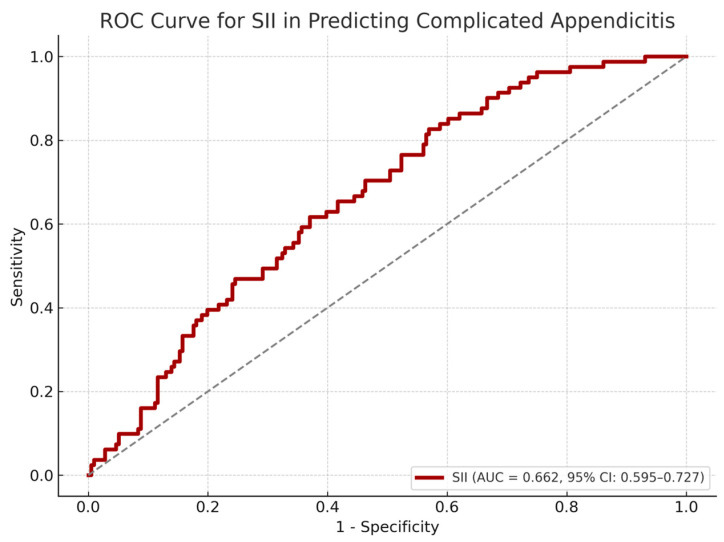
ROC curve for systemic immune-inflammation index in detecting complicated appendicitis among pediatric patients.

In a subgroup analysis of appendicitis patients, SII was significantly higher in those with complicated disease (median SII: 3213.4 vs. 1804.6, *p* < 0.001). As detailed in Table 3, SII demonstrated moderate discriminative power in identifying complicated cases, with an AUROC of 0.66 (95% CI: 0.60–0.73). At a cut-off of >1774.88, SII yielded 83% sensitivity but only 43% specificity. The ROC curve for this analysis is shown in Figure 2. While the high sensitivity supports the use of SII as a rule-out tool, its limited specificity underscores the need for complementary clinical and radiologic evaluation.

## 4. Discussion

Our study demonstrates that the SII possesses excellent discriminative ability in the diagnosis of acute appendicitis in pediatric patients. With an area under the curve (AUC) of 0.95 (95% CI: 0.92–0.97) and an optimal threshold of >624, the SII yielded a sensitivity of 91% and a specificity of 88%, supporting its utility as a rapid, cost-effective, and readily accessible biomarker in the evaluation of acute surgical conditions.

The success of treatments is often predicted by evaluating numerous factors, and significant effort is devoted to identifying the most suitable patient for the planned therapeutic approach. Among these factors, laboratory parameters have gained considerable attention as predictive, diagnostic, and severity-indicating biomarkers, making biomarker studies a consistently popular area of research. While new biomarkers meet many needs, the time it takes to work affects the decision for an acute surgical intervention. In addition, the fact that these new biomarkers are quite expensive has led to alternatives. Complete blood count is a very simple, cheap, and rapid test that provides important data. Changes in lymphocyte subgroups reflect the response of the immune system to the inflammatory condition and show the critical role of leukocyte inflammation. Neutrophils are the primary effector cells of the immune system and show phagocytosis and apoptotic activity through the synthesis of pro-inflammatory molecules [12]. Lymphocytes are specific inflammatory mediators that play a critical role in the regulation of humoral and cellular immune responses. Platelets are among the early inflammatory markers due to their effects on modulation of endothelial permeability and regulation of neutrophil and macrophage migration [13]. It has been reported that an increase in platelet count is strongly correlated with pro-inflammatory molecules and acute phase reactants. Lymphocytopenia indicates a deterioration in cellular immunity, while neutrophilia and thrombocytosis are accepted as indicators of systemic inflammatory response. In recent years, the Systemic Immuno-Inflammation Index (SII), which is formed by evaluating these three important immune-inflammatory parameters (neutrophil, lymphocyte, and platelet) together, has been increasingly accepted in clinical practice. SII offers the opportunity to evaluate inflammatory processes and the status of the immune system in a more comprehensive way. Studies have shown that SII may be used as a prognostic marker in various clinical conditions such as many types of cancer, acute appendicitis, ischemic stroke, and cardiovascular diseases [14,15,16,17,18]. SII stands out because of its higher predictive value, especially when compared to other inflammatory markers such as NLR and PLR. High SII values are generally associated with thrombocytosis, neutrophilia, and lymphopenia, and these findings indicate that the patient’s systemic inflammatory response is severe. Therefore, the use of SII in clinical practice may contribute to a better understanding of the inflammatory and immunological conditions of patients and to more effective planning of treatment strategies.

Previous studies in adult populations have shown that SII surpasses other inflammatory markers—such as neutrophil-to-lymphocyte ratio (NLR) and platelet-to-lymphocyte ratio (PLR)—in predicting complicated appendicitis, particularly in the context of perforation [8]. Similarly, pediatric emergency studies have emphasized the value of SII not only in diagnosing appendicitis but also in anticipating complications such as perforation and intra-abdominal spread [9].

In the study by Telafarlı et al., SII was reported to be significantly elevated in both acute and complicated appendicitis and was found to correlate positively with well-established clinical scoring systems [10]. Interestingly, while SII demonstrated greater accuracy in diagnosing acute appendicitis, NLR (neutrophil-to-lymphocyte ratio) was more effective in predicting complicated cases. Similarly, in a recent study by Altuğ et al. [17], SII was shown to effectively distinguish both acute and complicated appendicitis in pregnant patients. The present findings align with previous studies in pediatric populations, where ratios such as NLR and PLR have demonstrated a moderate ability to discriminate between uncomplicated and complicated appendicitis. For instance, Anastasakis et al. reported that NLR values > 7.92 and PLR values > 180.57 could predict complicated appendicitis with reasonable sensitivity and specificity (62.5% and 74.2% for NLR; 61.1% and 68.9% for PLR, respectively) [19]. Interestingly, these indices also showed a strong linear correlation, suggesting that combined evaluation may further improve diagnostic accuracy. Although SII has not been directly compared in this context, the shared inflammatory components underpinning these indices support their concurrent utility. This highlights the potential of combining SII with other hematologic markers to enhance clinical decision-making in ambiguous cases.

In recent years, interest has grown in evaluating the SII as a diagnostic and prognostic biomarker in pediatric appendicitis. However, pediatric-focused studies remain limited. Studies demonstrated the potential of SII to differentiate between simple and complicated appendicitis in children, although their work lacked robust cut-off analysis or external validation. More recently, Guo et al. reported that the combination of SII with the Pediatric Appendicitis Score (PAS) significantly improved the prediction of disease severity and surgical outcomes in children aged 5 years and older, achieving an AUC of 0.91 for severity assessment and 0.86 for prognosis prediction [20]. While their study emphasized the utility of combining clinical and laboratory parameters, our study addresses this gap by providing a large, age-homogeneous pediatric sample, assessing the standalone performance of SII, and identifying optimal SII thresholds for both acute and complicated appendicitis using ROC curve analysis.

What is particularly noteworthy is that SII consistently demonstrates strong discriminative power across diverse patient populations including adults, pregnant women, and children. This suggests its potential utility as a broadly applicable inflammatory biomarker, regardless of age or physiological status. However, slight variations in optimal cut-off values and predictive accuracy across studies emphasize the need for age- and context-specific validation. Our study contributes novel cut-off points and performance metrics tailored to pediatric patients, reinforcing the potential role of SII in clinical algorithms for early diagnosis and risk stratification in this population.

Elevated SII values may reflect more advanced or complicated stages of appendicitis. In contrast to conventional measures like CRP or total leukocyte count, SII concurrently reflects both pro-inflammatory activity (neutrophilia and thrombocytosis) and stress-induced immune suppression (lymphopenia). Despite its extensive application in pediatric appendicitis diagnosis, CRP has relatively mediocre diagnostic efficacy. Conversely, SII offers enhanced accuracy, perhaps minimizing superfluous imaging or surgical procedures in instances of confusing clinical presentations.

A key benefit of SII is its foundation in standard complete blood count (CBC) values, necessitating no supplementary tests or laboratory resources. This simplicity is particularly advantageous in resource-constrained environments. Nonetheless, specific constraints must be acknowledged: Hematological values in children fluctuate with age, complicating the establishment of universal cut-off standards. Moreover, concurrent infections or chronic inflammatory conditions may influence baseline SII levels and should be read with caution.

This study has several limitations. First, the age range of the study population was restricted to children between 5 and 15 years, which may limit the generalizability of the findings across the broader pediatric spectrum. Multicenter trials with age-stratified analyses are required to determine the clinical value of SII in standard pediatric practice. Second, established clinical scoring systems such as the PAS, the Alvarado Score, or the Appendicitis Inflammatory Response score were not evaluated into the diagnostic process as a nature of retrospective design. Unfortunately, our study’s retrospective design did not allow for the consistent retrieval of all clinical variables required for these scores. Integrating SII into current scoring systems could augment early diagnostic precision and boost patient outcomes.

## 5. Conclusions

In clinical practice, the integration of SII and NLR into diagnostic algorithms could enhance early detection and triage, particularly in settings where imaging is not immediately available. However, for predicting complicated cases, these biomarkers should be interpreted alongside clinical signs and radiological findings to avoid overdiagnosis or overtreatment. SII appears to be a valuable supplementary tool in diagnosing acute appendicitis in pediatric patients. When analyzed in conjunction with clinical assessment and imaging results, it may function as an essential instrument for early diagnosis, especially in pediatric patients exhibiting atypical or non-specific symptoms. However, its incorporation into clinical pathways must be preceded by additional validation via larger, prospective trials.

## Figures and Tables

**Table 1:** Baseline characteristics and laboratory findings in pediatric patients with group normal and appendicitis.

Variable	Group Normal (*n* = 100)	Group Appendicitis (*n* = 207)	*p*
Age (years)	9.00 (6.00–12.00)	10.00 (7.00–13.00)	0.055 ^a^
Sex (female/male)	25/75	89/208	0.342 ^b^
Duration of hospitalisation (days)	2.00 (1.00–2.00)	3.00 (3.00–7.00)	<0.001 ^a^
WBC (×10^3^/µL)	7.23 (6.29–9.44)	14.97 (11.84–18.46)	<0.001 ^a^
NEU (×10^3^/µL)	3.10 (2.63–4.14)	12.25 (9.28–15.42)	<0.001 ^a^
LYM (×10^3^/µL)	3.13 (2.49–3.90)	1.67 (1.26–2.54)	<0.001 ^a^
PLT(×10^3^/µL)	331.50 (285.75–387.25)	315.00 (262.00–363.00)	0.043 ^a^
Monocyte (×10^3^/µL)	0.54 (0.44–0.67)	0.92 (0.70–1.18)	<0.001 ^a^
Eosinophil (×10^3^/µL)	0.13 (0.09–0.23)	0.02 (0.00–0.08)	<0.001 ^a^
Basophil (×10^3^/µL)	0.03 (0.02–0.04)	0.03 (0.01–0.04)	0.696 ^a^
Hemoglobin	13.95 (13.20–15.20)	13.80 (12.90–14.90)	0.732 ^a^
HTC	40.85 (39.18–43.28)	41.40 (38.30–44.00)	0.825 ^a^
CRP (mg/L)	1.12 (0.43–2.24)	32.90 (10.80–95.07)	<0.001 ^a^
NLR	1.08 (0.71–1.35)	7.64 (4.32–11.63)	<0.001 ^a^
PLR	103.55 (81.07–134.28)	184.02 (116.03–254.14)	<0.001 ^a^
SII	356.27 (245.16–462.18)	2218.43 (1262.05–3788.45)	<0.001 ^a^

All values given numbers, mean ± standard deviation or median (IQR), WBC: white blood cell, CRP: C- reactive protein, PLT: platelets, NEU: neutrophil, LYM: lymphocyte, HTC: hematocrit, PLR: platelet/lymphocyte ratio, NLR: neutrophil/lymphocyte ratio, SII: systemic immune inflammation index, ^a^ Mann–Whitney U test, ^b^ Chi-square test.

**Table 2:** Diagnostics accuracy of inflammatory parameters to predicting acute appendicitis in pediatrics.

Parameter	AUROC (95% CI)	*p*	Youden’s Index	Criterion	Sensitivity	Specificity	PPV (95% CI)	NPV (95% CI)
NLR	0.96 (0.94–0.98)	<0.001	0.83	>2.52	0.89	0.94	0.98 (0.95–0.99)	0.73 (0.65–0.80)
PLR	0.77 (0.72–0.81)	<0.001	0.48	>155.93	0.62	0.86	0.93 (0.88–0.96)	0.43 (0.36–0.50)
SII	0.95 (0.92–0.97)	<0.001	0.79	>624.14	0.91	0.88	0.96 (0.93–0.98)	0.76 (0.67–0.83)

AUROC: area under the receiver operating characteristic, SII: systemic immune-inflammation index, PPV: positive predictive value, NPV: negative predictive value.

**Table 3:** Diagnostics accuracy of inflammatory parameters to predicting complicated vs. uncomplicated acute appendicitis in pediatrics.

Parameter	AUROC (95% CI)	*p*	Youden’s Index	Criterion	Sensitivity	Specificity	PPV (95% CI)	NPV (95% CI)
NLR	0.61 (0.54–0.68)	<0.001	0.19	>8.31	0.58	0.61	0.35 (0.28–0.44)	0.79 (0.72–0.85)
PLR	0.65 (0.57–0.72)	<0.001	0.26	>209.54	0.58	0.68	0.40 (0.32–0.49)	0.81 (0.74–0.86)
SII	0.66 (0.60–0.73)	<0.001	0.26	>1774.88	0.83	0.43	0.35 (0.28–0.42)	0.86 (0.78–0.91)

AUROC: area under the receiver operating characteristic, SII: systemic immune-inflammation index, PPV: positive predictive value, NPV: negative predictive value.

## Data Availability

The raw data supporting the conclusions of this article will be made available by the authors on request.

## Abbreviations

The following abbreviations are used in this manuscript:

AA	Acute appendicitis
CRP	C-reactive protein
NLR	neutrophil-to-lymphocyte ratio
PAS	pediatric appendicitis score
PLR	platelet-to-lymphocyte ratio
SII	systemic immune-inflammation index
WBC	white blood cell count
